# A Theoretical Framework of Implicit Care Rationing in Australian Long‐Term Aged Care Settings: A Straussian Grounded Theory Study

**DOI:** 10.1155/jonm/8676600

**Published:** 2026-04-29

**Authors:** Xinxia Wang, Kasia Bail, Victoria Traynor

**Affiliations:** ^1^ School of Psychology, Faculty of Science, Medicine and Health, University of Wollongong, Wollongong, New South Wales, Australia, uow.edu.au; ^2^ Faculty of Health, University of Canberra, Canberra, Australian Capital Territory, Australia, canberra.edu.au; ^3^ School of Health, University of Sunshine Coast, Sippy Downs, Queensland, Australia, health.qld.gov.au

**Keywords:** care delivery arrangement, decision-making process, grounded theory, healthcare rationing (MeSH), implicit care rationing, long-term aged care settings, missed care, prioritisation

## Abstract

**Background:**

Implicit care rationing (ICR), where care delivery is strategically arranged by frontline healthcare professionals under resource constraints, is widely acknowledged across healthcare settings. However, its theoretical conceptualisation remains underdeveloped in the context of long‐term aged care (LTAC) settings worldwide.

**Aim:**

This study aimed to develop a theoretical framework of ICR in Australian LTAC by exploring the actual care decision‐making processes of hands‐on healthcare staff through the lens of Donabedian’s model.

**Methods:**

Using a Straussian grounded theory approach, data were collected in two phases. Phase 1 involved developing a preliminary framework through in‐depth interviews with 26 registered nurses, clinical managers and personal care workers. Phase 2 validated and refined the framework through focus group discussions with another 18 healthcare staff in the above roles. A combination of online and in‐person strategies was used for participant recruitment and data collection. Constant comparative analysis was conducted concurrently with data collection and supported by an iterative review of relevant literature.

**Results:**

The developed theoretical framework of ICR unravels the relevant influencing factors, the cognitive pathway and the prompt care delivery outcomes that are evident in Australian LTAC. This framework conceptualises ICR as a dynamic, shift‐level decision‐making process concerned with the order and completeness of care delivery. This cognitive process is driven by the intuitive assessment of healthcare staff of both external and internal impact factors, guided by a risk‐averse, consequence‐focused mindset and trade‐offs between person‐centred and task‐focused care approaches.

**Conclusions:**

The developed theoretical framework enhances academic consistency and rigour through an evolutionary reconceptualisation of ICR grounded in strategic healthcare resource allocation. It clarifies the relationship between ICR and missed care, challenging their interchangeable use in previous research. This framework also offers practical insights to inform workforce strategies, measurement development and targeted interventions, ultimately contributing to improved care quality and safety in Australian LTAC.

## 1. Introduction

Healthcare rationing is a classic strategy to distribute limited healthcare resources, with its origins traceable to the post‐World War II decades and its introduction as a Medical Subject Heading in 1989 [1]. The adoption of rationing reflects the reality that healthcare resources, including funding, workforce, equipment, treatments and medications, are finite, yet the demand often exceeds capacity regardless of a country’s wealth or the sophistication of its healthcare system [2]. The aim of healthcare rationing is to achieve a balance between equity, efficiency and sustainability in resource allocation by ensuring fair access to essential care and maximising the overall benefit derived from limited resources [2]. In practice, rationing occurs across all levels of stakeholders in a healthcare system, from government bodies determining which services are funded [3], through healthcare professionals deciding who receives certain services [4], to clients making decisions when faced with out‐of‐pocket costs [5]. Healthcare rationing is typically categorised as either explicit or implicit in the context of the decision makers, criteria to be considered and levels of operation.

Explicit rationing involves formal policies, guidelines or frameworks grounded in principles such as cost‐effectiveness, population health needs and service delivery priorities [3]. It is often preferred by policymakers and healthcare organisations for its transparency, consistency and accountability to public scrutiny. A prominent example of explicit rationing is the value framework and roadmap for COVID‐19 vaccine allocation issued by the World Health Organization [6], which prioritised limited vaccine supplies to frontline healthcare professionals and older people who needed them most. Implicit rationing, in contrast, occurs informally in everyday clinical practice, depending on healthcare professionals’ educational background, heuristic habits learnt from working experience and tacit team norms [4]. Examples of implicit rationing involve nurses omitting assessments after delivering treatments or physicians postponing nonurgent imaging orders because of a full schedule [4]. While implicit rationing allows flexible, situationally tailored decisions, it is unstandardised and often invisible to consumers and audit bodies. Explicit rationing and implicit rationing have been mostly studied in acute clinical settings, since they require a delicate balance between effectively managing clients’ life‐threatening conditions and sustainably allocating limited healthcare resources.

Although much of the existing literature has examined healthcare rationing in acute clinical settings, increasing attention is now turning to aged care as widening demand–supply gaps intensify under the global ageing trend. On the one hand, the older population’s healthcare demands are elevated because they are living with more complex and severe health issues for longer periods of time [7]. On the other hand, constrained budgets and competing policy priorities have made it challenging for governments to manage escalating demands, prompting the adoption of explicit rationing strategies to allocate stretched healthcare resources more effectively to older people [2]. Countries including Australia [8], the United States [9] and the United Kingdom [10] use means‐testing methods to determine eligibility for aged care services and establish the level of care and subsidies provided. Means‐testing methods lead to increased out‐of‐pocket payments for older people and shift part of the cost burden to older people with greater financial means [3]. However, this approach also limits the resources available to aged care providers by restricting public budgets. Many providers struggle to recruit and retain healthcare staff due to low wages and poor working conditions, contributing to persistent workforce shortages [11].

Despite workforce shortages being prevalent across all aged care services, this condition is comparatively pronounced in the long‐term aged care (LTAC) sector, as most older people living in LTAC settings rely heavily on consistent and high‐quality clinical and personal care provided by professional healthcare staff. In Australia, 8400 registered nurses (RNs) and 13,300 personal care workers (PCWs) are required to provide care to older people living in LTAC settings, with an average of 215 nursing minutes per day and at least one RN onsite and on duty around the clock [12]. In the United Kingdom, 13,500 vacancies in healthcare staff were identified in nursing homes [13]. In the United States, 46% of nursing homes are delaying admissions due to an insufficient workforce [14]. The substantial workforce shortages, together with time pressure, heavy documentation requirements, inadequate clinical supplies, poor teamwork and limited supervisory support [15], stretch current healthcare staff working in LTAC settings. As a result, staff are often forced to adopt implicit rationing approaches to deliver care services as effectively as possible [16].

In the current literature, implicit care rationing (ICR) is commonly defined following Schubert [17] as ‘the withholding of or failure to carry out necessary nursing measures for patients due to lack of nursing resources’. This definition strongly emphasises healthcare professionals’ decisions about care omissions, whether partial or complete. Consequently, ICR has often been operationalised as a form of care omission, and related terms such as ‘missed care’, ‘care left undone’ and ‘unfinished care’ are frequently used interchangeably with ICR [18]. Given the high prevalence of care omission and its significant potential to cause detrimental outcomes [19], ICR has been widely examined in LTAC settings in countries such as Australia [20], the United States [21] and the United Kingdom [22]. A clear relationship between the incidence of care omissions and poor care quality and safety was determined, as each omitted care activity removes a protective layer that would otherwise prevent falls, pressure injuries, infections, pain crises and hospital readmissions [18].

However, Schubert’s definition of ICR does not adequately capture the broader strategic nature of rationing, whereby care is not merely omitted but actively prioritised and sequenced by healthcare professionals in response to resource constraints. In other words, this definition overlooks the interdependent decision‐making process of determining whether and when to provide or withhold services under conditions of competing priorities and finite resources. Recent studies [23, 24] articulated this relationship, indicating that the prioritisation initiates a temporary ordering of care tasks which can be a precursor to the omission of low‐priority tasks with consequent risks to client safety. This highlights a significant theoretical gap in the conceptualisation of ICR, stemming from the lack of holistic consideration of the interconnectedness within care delivery arrangements.

Given this limited conceptualisation of ICR, no studies to date have adequately explained the cognitive processes underpinning this phenomenon in LTAC settings. Although a recent study [25] revealed ICU nurses’ decision‐making processes behind missed nursing care, important distinctions between LTAC and acute clinical settings must be acknowledged, including differences in the primary healthcare objectives, the nature of care and the workforce characteristics. Rather than intensive nursing interventions for curing diseases, personal care is an important component in addition to clinical care in LTAC settings, emphasising person‐centredness for the overall well‐being of older people [26]. Additionally, the ratios of RNs to clients are quite low in LTAC settings, with the majority of care services being delivered by PCWs who receive less training and education compared to nurses, whereas RNs dominate service delivery in hospitals [27]. These aspects can significantly impact the cognitive processes and outcomes of care delivery arrangements, underscoring a specific concern about ICR in the context of LTAC. Moreover, studies have suggested that better care delivery arrangements can optimise allocation of limited healthcare resources, promote care outcomes and reduce the incidence of adverse events [28]. Therefore, a thorough understanding of cognitive processes during ICR is of great value in improving care quality and safety in Australian LTAC settings.

To address this critical gap, the present study aimed to develop a grounded theory framework that captures the cognitive processes involved in ICR within Australian LTAC. Specifically, it sought to answer three research questions: (1) What factors influence the cognitive processing of ICR by hands‐on healthcare staff? (2) What is the actual cognitive pathway of ICR at the shift level? (3) What are the decision‐making outcomes resulting from ICR?

## 2. Method

**FIGURE 1:**
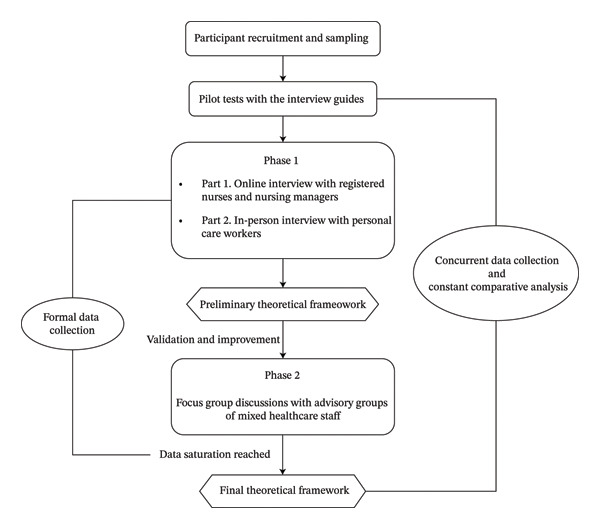
Overall research design for the theoretical framework development.

This study adopted a Straussian grounded theory (SGT) approach [29] to develop a theoretical framework of ICR based on the perceptions of hands‐on healthcare staff working in Australian LTAC settings. Data collection proceeded in two phases, including in‐depth interviews that generated an initial framework in Phase 1 and the focus group discussions to validate and refine the framework to its final form in Phase 2. Data analysis was conducted concurrently with data collection, using a constant comparative approach whereby newly collected data were continuously compared with existing codes, categories and the provisional theoretical framework developed from earlier data and relevant literature. This study was approved by the Human Research Ethics Committee of the University of Wollongong, Australia (ethics number: 2024/07). The Standards for Reporting Qualitative Research (SRQR) Guideline [30] and the Consolidated Criteria for Reporting Qualitative Research (COREQ) checklist [31] (Supporting Information 1) were applied to ensure transparency and comprehensiveness in reporting. An overview of the research design is presented in Figure 1.

### 2.1. Methodology

Grounded theory is a classic qualitative methodology for generating theories that highlight behaviour, develop perspectives and evolve through category formation, which is particularly relevant when existing literature on a topic is scarce or superficial [32], as is the case with ICR in LTAC. This study adopted the SGT approach developed by Strauss and Corbin [29], which is rooted in pragmatism, symbolic interactionism and constructivism, incorporating both inductive and deductive reasoning [33]. Other than generating theory purely from empirical data, SGT encourages researchers’ active interpretation in shaping theory via the early integration of literature evidence. The combination of empirical data and prior knowledge improves theoretical sensitivity throughout data collection and analysis, amplifying the researchers’ ability to recognise, interpret and give meaning to relevant data in building a conceptually rich and academically robust theoretical framework [34]. Additionally, SGT employs a systematic methodology, characterised by its structured, iterative and reflexive approach to theory development, ensuring analytical transparency with a full consideration of the influence of the researcher’s perspective [33]. Therefore, SGT proved to be exceptionally effective in generating detailed, grounded insights into the intricate cognitive processes of ICR in LTAC settings, aligning closely with the purpose of this study.

### 2.2. Theoretical Foundation

Donabedian’s model [35] was applied to articulate the researchers’ understanding of ICR and inform the development of interview guides as well as data analysis. Donabedian’s model conceptualises care services in three categories as ‘structure’, ‘process’ and ‘outcome’, describing the factors that affect the context of care delivery, the interactional aspects of care delivery and the effects of delivered care on clients, respectively. This study drew a conceptual alignment with Donabedian’s model by positioning ICR as the process rather than the outcome under investigation. In the current study, ICR was considered as a decision‐making process wherein healthcare staff navigate strategic care delivery arrangements in response to structural factors such as multiple resource constraints. These decisions can result in varying care outcomes, including care being delivered or omitted. While Donabedian’s model primarily emphasises outcomes as changes in clients’ health status, this study adopts a proximal lens, conceptualising care arrangement decisions as intermediate outcomes that mediate downstream effects on the safety, well‐being and care quality of older people living in LTAC settings [36]. This modified application preserves the integrity of Donabedian’s evaluative triad while enabling a theory‐driven exploration of ICR’s processual character.

### 2.3. Participant Recruitment and Sampling

This study recruited clinical managers, nurses and PCWs working in Australian LTAC. These frontline healthcare staff were considered most likely to possess empirical knowledge of ICR, given their routine involvement in making care delivery decisions under time and workforce constraints [37]. To participate, clinical managers were required to have at least 1 year of experience in a managerial or supervisory position in Australian LTAC settings, serving as clinical nursing managers, personal care managers and other leadership roles directly involved in care planning and oversight. Nurses, including RNs and enrolled nurses (ENs), were required to possess a minimum of 2 years’ experience in Australian LTAC settings, with expertise in gerontological nursing. PCWs were eligible if they had a minimum of 1‐year experience working in LTAC settings and held at least a Certificate III in Ageing Support (CHC43015) [38] or an equivalent recognised vocational qualification in Australia. Additionally, all participants were required to communicate fluently in English and consent to audio or video recording during interviews or discussions.

Participant recruitment and sampling were conducted across two phases using a combination of purposive and convenience sampling strategies. In Phase 1, individual healthcare staff were invited to participate in in‐depth interviews. Nurses and clinical managers were recruited via online channels, while PCWs were approached in person within an LTAC setting. With authorisation from Victoria Traynor and Kasia Bail, the copresidents of the Gerontological Alliance of Nurses Australia (GANA), recruitment materials were disseminated through GANA’s LinkedIn post and membership mailing list, targeting nurses and clinical managers nationwide. Recruitment information was provided in the form of a digital poster, which included hyperlinks and QR codes directing potential participants to the participant information sheet (PIS) and the consent form (CF) hosted on Qualtrics. Recruitment was confirmed upon receipt of completed CFs through the Qualtrics platform. Subsequently, Xinxia Wang, another researcher, scheduled interviews by emailing polling links and confirming session times based on participant availability.

Given GANA’s membership largely comprises nurses, PCWs were recruited separately by Xinxia Wang through on‐site engagement at a local LTAC setting operated by Warrigal in Wollongong, New South Wales. Following institutional approval for data collection, recruitment materials, including posters, PISs and CFs, were displayed in staff common areas such as the canteen and nursing stations. In addition, Xinxia Wang approached PCWs directly in the corridors using convenience sampling to invite eligible and available individuals to participate.

In Phase 2, nurses, clinical managers and PCWs were recruited to participate in mixed‐role advisory groups for focus group discussions. Leveraging professional networks within the aged care sector, Victoria Traynor disseminated recruitment materials to multiple aged care providers across Australia. Two settings consented to participate: Immanuel Gardens Aged Care, operated by Lutheran Services in Buderim, Queensland, and Uniting Farmborough residential aged care home in Unanderra, New South Wales. Recruitment materials were forwarded to facility managers, who assisted in identifying and inviting eligible staff to join the advisory groups on behalf of the research team.

### 2.4. Data Collection

Data were collected between August 2024 and June 2025, with Phase 1 conducted from August 2024 to April 2025 and Phase 2 taking place in May and June 2025. Xinxia Wang, a female PhD candidate in nursing with a research focus on LTAC since her Master’s degree, conducted all data collection. To prepare for these roles, Xinxia Wang completed postgraduate coursework and practical training in qualitative research methods, including interview techniques, focus group facilitation and NVivo‐based data analysis. This background provided in‐depth familiarity with LTAC practice and the challenges faced by healthcare staff, which helped to build rapport with participants and to ask contextually relevant follow‐up questions during interviews and focus group discussions. To address this, Xinxia Wang made brief reflexive notes after each interview and focus group, documenting prior assumptions, emotional reactions and initial impressions, which were later incorporated into the analytic memos to support a more critical reading of the data.

In general, each data collection session comprised four steps: obtaining informed consent from participants, collecting demographic information, conducting individual interviews or group discussions and distributing shopping vouchers as tokens of appreciation. All nurses and managers who participated in Phase 1, as well as all advisory group members in Phase 2, received a voucher valued at AUD $50. PCWs who participated in Phase 1 were entered into a lottery draw (20% chance) for a voucher at the same value. At the beginning of each session, following a brief introduction between Xinxia Wang and the participant(s), Xinxia Wang would explain the study’s background, purpose and design as written in the PIS. During all sessions, field notes were taken to capture nonverbal cues, environmental interruptions, external influences and group dynamics where applicable.

Phase 1 data collection involved two parts: semistructured, online interviews with nurses and clinical managers and structured, in‐person interviews with PCWs. The initial interview guides for both groups were drafted by Xinxia Wang based on a review of relevant literature and her professional experience as an RN. These guides were subsequently refined with input from her supervisors, who have substantial experience in qualitative research. Pilot testing was conducted with one RN and one PCW currently working in LTAC settings. Based on their feedback, the guides were fine‐tuned to enhance clarity and comprehensibility (see Supporting Information 2).

For nurses and clinical managers, informed consent was obtained via Qualtrics. Upon returning their consent forms, participants received a hyperlink to a Qualtrics‐hosted demographic survey, collecting data on age, gender, highest level of education, nursing qualification, current primary roles and responsibilities and years of experience in their current roles. Completion of the survey was required prior to the interview to enable the interviewer to develop a preliminary understanding of each participant. Interviews were conducted via Microsoft Teams or Zoom, depending on participant preference, and were video recorded. Each session lasted approximately 1 h.

For PCWs, informed consent was obtained either through written consent forms or verbally, acknowledging the time constraints inherent in their work routines. Verbal consent was audio‐recorded at the beginning of the session and included the interviewer’s explanation of the study and the participant’s verbal agreement to take part. Demographic data were collected via a paper‐based survey. Structured interviews were conducted in quiet, convenient locations, such as the staff canteen after formal lunch breaks or a vacant meeting room. To reduce disruption to normal workflow, sessions were limited to 30 min. Audio recordings were made using Microsoft Teams with the video function disabled.

Phase 2 comprised four in‐person, on‐site focus group discussions with advisory groups of mixed‐role healthcare staff. Two sessions were held at each participating setting, each lasting approximately 1 h. All discussions were conducted in private meeting rooms with closed doors and no external interruptions. At the start of each session, participants provided written consent and completed the same demographic survey used in Phase 1. The draft theoretical framework developed from Phase 1 was presented using slides and printed handouts, which included the proposed categories, codes and anonymised quotations. Participants were first asked to provide written feedback on the framework’s relevance, clarity, comprehensiveness and any perceived redundancies. Group discussions followed, during which participants were encouraged to reflect and share their perspectives. These sessions were audio‐recorded using Microsoft Teams with video disabled.

### 2.5. Data Analysis

Data analysis was conducted in two sequential stages: the development of a preliminary theoretical framework based on the analysis of Phase 1 interview data, followed by refinement and validation using data from Phase 2 focus group discussions. Guided by an iterative and constant comparative approach conducted concurrently with data collection, the analysis followed the structured coding sequence proposed by Strauss and Corbin [29], which includes open coding, axial coding and selective coding. Prior to analysis, transcripts auto generated by Microsoft Teams or Zoom were carefully reviewed and cleaned by cross‐checking against original video and audio recordings. These refined transcripts, supplemented with field notes, were uploaded into NVivo 14 for further coding.

Coding was jointly conducted by Xinxia Wang and her supervisor Victoria Traynor. Victoria Traynor is a professor of nursing and an experienced gerontological nursing researcher with more than three decades of quantitative and qualitative research and education experience in the context of Australian LTAC. Xinxia Wang’s close involvement in data collection meant she brought detailed, contextual knowledge of each interview and focus group to the analytic process, while also being at risk of over‐identifying with participants’ accounts. Victoria Traynor contributed a more distanced disciplinary and policy perspective, drawing on extensive experience in LTAC research and grounded theory methodology to question early interpretations and consider alternative explanations for emerging categories. Xinxia Wang and Victoria Traynor met regularly in scheduled semimonthly meetings to discuss coding decisions, resolve discrepancies and ensure analytic coherence.

During the open coding phase, preliminary codes were generated inductively based on the meanings emerging from the data. This involved identifying significant words or phrases and assigning either conceptual or in vivo labels. Conceptual codes reflected the inferred meanings of incidents, actions and interactions, while in vivo codes preserved participants’ exact words to retain their voices and lived experiences. The initial relationships identified among these codes informed the axial coding phase, where codes were grouped into broader, higher‐order categories. In the axial coding phase, core categories began to emerge and the dynamic relationships between them were explored and refined. Categories were continuously adjusted until data saturation was achieved when existing categories were deemed sufficiently explanatory. Saturation was monitored at the level of these higher‐order categories across participant roles and was considered reached when additional empirical data only confirmed and deepened previously identified patterns and relationships. In the final stage, selective coding categories were further refined and integrated into a coherent theoretical framework. During this phase, each category was examined in turn, with other categories added, merged or discarded to build a concise and meaningful interpretive model. Coding continued until the theoretical framework was validated by advisory groups, with no further modifications required.

Throughout the analysis, relevant literature was reviewed continuously to enhance theoretical sensitivity, serving not to predefine codes from the data but to inform concepts’ extraction, to identify concepts which occurred in both data and the literature and to differentiate novel findings specific to this study [33]. This process also supported conceptual precision and minimised interpretive bias by referring to well‐defined concepts in previous literature. Additionally, analytical and reflective memos were written throughout the study to support theory construction and promote methodological transparency [34]. These included memos documenting the rationale for coding decisions, category development, comparative reflections across participant groups, saturation checks and diagrammatic revisions. Reflexive memos further facilitated critical examination of how researchers’ disciplinary backgrounds, assumptions and professional experiences may have shaped data interpretation and participant engagement.

### 2.6. Rigour and Trustworthiness

The methodological rigour and theoretical trustworthiness of the developed framework were ensured by adhering to the four criteria proposed by Lincoln and Guba [39]: credibility, transferability, dependability and confirmability.

Credibility, concerning the truthfulness of data and interpretations, was achieved through data triangulation and member checking. Triangulation was facilitated by engaging healthcare staff with varied roles and perspectives through individual interviews and focus group discussions and by incorporating field notes and memos to enrich interpretation. Member checking occurred during Phase 2 advisory group discussions, in which preliminary categories and the draft framework were presented to participants for feedback on their clarity and resonance, and their comments were used to clarify concepts and refine category labels. In addition, findings were reviewed collaboratively by the two coders and crosschecked with the coauthor during bimonthly meetings as the analysis advanced. Transferability, which refers to the extent to which the findings apply to other contexts or groups, was supported by recruiting participants from different LTAC settings across Australia. Given that the available healthcare resources and applied regulations differ between regions and aged care providers, developing and validating the theoretical framework with healthcare staff from geographically and organisationally diverse settings enhanced its applicability under varying conditions. Detailed descriptions of participants’ sociodemographic characteristics were provided as findings to enable readers to judge the applicability of the framework to their own contexts.

Dependability concerns the consistency of the research process, which was addressed through systematic coding practices and a well‐documented audit trail. The two coders held regular meetings to reconcile discrepancies and ensure coherence, while the progression of the analytical framework was traced through NVivo’s auto‐saved project logs and detailed memo writing. Confirmability requires that the findings are grounded in the participants’ data rather than shaped by researcher bias. To support data‐grounded analysis, in vivo codes were used during the open coding phase to preserve participants’ language, supplemented by keeping analytical and reflective memos to critically examine the impacts of interpretations by the researchers. Advisory groups’ validation further reinforced the confirmability of the framework by affirming its relevance to everyday practice.

## 3. Findings

### 3.1. Participants

A total of 44 healthcare staff participated in the study with detailed demographic information of participants being presented in Table 1.

**TABLE 1:** Demographic information of participants (*n* = 44).

Demographic variables	Phase 1 (*n* = 26)		Phase 2 (*n* = 18)	
	RNs and managers (*n* = 18)	PCWs (*n* = 8)	Group 1 (*n* = 8)	Group 2 (*n* = 10)
Age range (years)				
20 to 29	3	2	2	3
30 to 39	3	2	2	3
40 to 49	4	1	1	1
50 to 59	6	3	2	3
60 and above	2		1	
Gender				
Female	15	6	8	8
Male	3	2		2
Highest education level				
Vocational qualification		4	4	4
Diploma‐level qualification		2	1	
Bachelor’s degree	9	2	3	4
Master’s and above	9			2
Nursing qualification				
RN	16		4	5
PCW		8	4	5
Others	2			
Current primary role				
Hands‐on healthcare staff	9	8	7	8
Management/supervision	9		1	2
Working experience in current primary role (years)				
Hands‐on healthcare staff	2 to 35	3 to 13	3.5 to 40	2 to 31
Management/Supervision	3 to 10		2	3.5 to 5

Abbreviations: PCW, personal care worker; RN, registered nurse.

Phase 1 included 26 participants, including 18 RNs and clinical managers who participated in online interviews with an average duration of 55.8 min and eight PCWs who participated in in‐person interviews with an average duration of 26.3 min. During interviews with three RNs and one PCW, occasional background conversations and movements occurred but did not interfere with the data collection. Two RNs dropped out from the study after returning their consent forms yet did not respond to subsequent invitations for scheduling interviews. No repeated interview was conducted, and interview transcripts were not returned to participants for comment. For drafting the preliminary theoretical framework, no new elements emerged after the interviews with the 16^th^ participant in the group of RNs and managers and the 6^th^ PCW, with the additional interviews being conducted to confirm data saturation. Participating RNs and managers came from 12 LTAC providers located across four regions, including four in New South Wales, one in the Northern Territory, one in South Australia and two in Western Australia.

In Phase 2, 18 participants formed two advisory groups. Group 1 included eight participants from Immanuel Gardens Aged Care, while Group 2 comprised 10 participants from Uniting Farmborough Unanderra. Two sessions of focus group discussions were conducted on each site with each session, involving four participants of Group 1 and five participants of Group 2, respectively. Each session lasted an average of 62.5 min. By analysing the returned feedback forms and the discussion transcripts, no new elements emerged that warranted the addition of new codes or categories, nor were there suggestions to adjust the existing inter‐ or intra‐relationships between codes and categories. Instead, the advisory groups expressed a strong endorsement of the drafted framework, emphasising its comprehensiveness and truthfulness in depicting their daily care delivery experiences under resource constraints. Given a deep resonance with the framework’s existing codes and categories, they also shared vivid examples of ICR during discussions to prove and enrich the understanding of the concepts and structure. The discussion transcripts were not returned to participants or the managers who assisted with recruitment.

To ensure anonymity in presenting findings with participants’ quotations, each Phase 1 participant was assigned a numerical code based on their professional role. RNs were labelled RN 1 to RN 9, nurse managers as NM 1 to NM 9 and PCWS as PCW 1 to PCW 8. Participants in Phase 2 were anonymised using group number, session number and seat number during data collection, formatted as G1‐1‐1 to G1‐2‐4 for Group 1 and G2‐1‐1 to G2‐2‐5 for Group 2.

### 3.2. Theoretical Framework of ICR in Australian LTAC

This study conceptualised ICR in Australian LTAC settings as a decision‐making process characterised by healthcare staff’s cognitive pathway of ‘prioritisation–delegation/reprioritisation/postponement–relinquishment’ under resource constraints. This cognitive process directed both immediate individual and collaborative care delivery arrangements at the shift level. Staff decisions were influenced by a combination of external, objective factors and internal, subjective considerations, with an overarching focus on balancing efficiency with the quality and safety of care. The outcomes of these decision‐making processes were reflected in the order and completeness of care delivery.

#### 3.2.1. Factors Related to ICR in Australian LTAC

Participants identified several factors related to ICR in LTAC settings, substituted by explaining how these factors would influence the decision‐making process and outcomes of care delivery arrangements. These factors were divided into two dimensions, including external and objective factors as well as internal and subjective factors.

##### 3.2.1.1. External and Objective Factors Related to ICR in Australian LTAC

Five categories were identified as the external and objective factors that could impact healthcare staff’s decision‐making processes and outcomes of ICR, including workforce‐related challenges, workflow disruptions, operational attributes, systemic and contextual influence and resident‐related factors (Table 2).

**TABLE 2:** External and objective factors.

Categories	Codes	Subcodes
Workforce‐related challenges	Workforce shortages	Staff absence from rosters
		Workforce inadequacy despite meeting staffing ratios
	Workforce instability	Inappropriate allocation of junior team members
		Temporary allocation of clinical agency staff
		Inconsistent workforce
	Inflexible staffing	Overwhelmed workforce during peak hours
		Workforce depletion due to fluctuations

Workflow disruptions	Unexpected activities	Care‐related activities
		Noncare‐related activities
	Competing roles and responsibilities	Responsibilities as hands‐on care staff
		Multiple roles in a shift
	Collaboration difficulties	Coworkers’ resistance
		Limited support from allied health
	Leadership challenges	Dilemma of young leaders
		Uncooperative workforce
	Communication breakdowns	Between staff and residents
		Among healthcare staff

Operational attributes	Shift type	Morning shift, afternoon and night shift
	Facility characteristics	Facilities’ size
		Geographic locations
		Facilities or units’ procedures and protocols
	Organisation’s orientation	Financial‐focused
		Metric‐focused
	Workplace culture	Hierarchical culture
		Unsafe psychological environment
		Divisive culture
	Infrastructure and supply limitations	

Systemic and contextual influences	Public health crisis	
	Regulations, standards and policies	
	Inadequate funding and resources	
	Inadequate healthcare information shared between settings	

Resident‐related factors	Residents’ attitude towards ICR	Flexible with care delivery arrangements
		Stick to a regular routine
	Residents’ care needs	High needs
		Low needs
	Residents’ preference	Protect autonomy
		Familiarities and trustiness
	Families’ engagement	Negative complaints
		Provide support as informal carers

###### 3.2.1.1.1. Workforce‐Related Challenges

Workforce‐related challenges were the most frequently mentioned factors triggering the adoption of ICR, which manifests across three aspects as workforce shortages, workforce instability and inflexible staffing. Workforce shortages were prevalent in daily practice as healthcare staff were absent from rosters for reasons like sick leave yet without backfill to maintain the staffing ratios.

*You are meant to have five care workers on a shift, but only three or four turn up because someone called in sick and there is no one to replace them. In that case, you will definitely see care being rationed.* (NM 6)

Participants also noted that, even when the mandated staffing ratios were met, workforce inadequacy persisted. This was attributed to the fact that these ratios were established based on a pragmatic assessment of the aged care sector, where chronic workforce shortages driven by longstanding recruitment and retention challenges remain unresolved.

*A lot of it is around the ability to recruit both personal carers and registered nurses. [……] Workforce recruitment and retention create a big impact on how we deliver services.* (NM 3)

Workforce instability was reflected in three key areas, including the inappropriate allocation of junior team members, temporary allocation of agency staff and an inconsistent workforce. Due to persistent challenges in recruiting experienced RNs, it is not unusual for a setting to assign a newly graduated RN, who lacks working experience in LTAC, as the sole RN on duty to comply with the 24/7 RN requirement. In the absence of adequate supervision and support, these entry‐level RNs were expected not only to deliver all medical and clinical care services but also to manage the delegation of overall workflow during their shift. As highlighted by NM 2, such arrangements could result in care delivery being ‘critically wrong’, underscoring the risks associated with inadequate workforce preparation and support.

*There are a lot of graduate RNs who come into our industry and do not have that support. They come in as a novice. Although they have the training and the knowledge, they don’t have that depth of experience, so they become very task-focused and just want to get through their shift.* (NM 9)

To meet mandated staffing ratios, LTAC settings often rely on agency staff to fill temporary workforce gaps, even when these individuals lack the appropriate skill sets for aged care and have no prior experience working in LTAC settings. Participants noted that many agency nurses primarily come from acute care backgrounds and often seek additional income through agency shifts in these settings (NM 2). These staff members frequently struggle to adapt their clinical strategies to the distinctive care delivery models of LTAC, which also impacts the arrangement of care delivery (NM 1).

While some agency staff may be regularly assigned to the same setting, they are typically regarded as transient members of the workforce rather than integrated team members. As such, they often lack a sustained understanding of residents’ conditions, needs and preferences. Similarly, the frequent rotation of regular staff between different areas within a setting further contributes to workforce inconsistency. This continuous reshuffling prevents staff from building familiarity with the residents they care for, thereby impeding their ability to make informed prioritisation decisions. As RN 5 remarked, a lack of consistent knowledge about residents compromises the ability to identify and prioritise care services that matter most to them.

*There is a very casualised workforce when you got different staff or agency staff coming on. There is a lot of problem if a nurse does not necessarily appreciate the risk of looking after 40 people without much knowledge about them.* (RN 2)

Inflexible staffing refers to situations where, although a shift may commence with the required number of staff on duty, the rigid nature of staffing arrangements fails to accommodate surges in workload during peak periods or respond to unexpected workforce depletion due to fluctuations such as emergencies, resident behavioural changes or sudden staff fatigue and illness. In such scenarios, no additional workforce is available to support overwhelmed team members. As a result, healthcare staff are compelled to engage in ICR to manage competing demands under constrained conditions.

*If a resident’s behaviour escalates to a bit of delirium for a urinary tract infection, somebody has to be taken off the floor to sit with her [… …]. That then puts the rest of the shift back because instead of having four care staff on, you only got three now.* (NM 5)

Overwhelming workloads can contribute to cognitive overload among healthcare staff, increasing the likelihood of unintentional omissions due to forgetfulness.

*Mary is supposed to have blood glucose level checked four times a day. But you get busy; you have someone with delirium and two dressings to do, and then you just forget to check Mary’s glucose level.* (RN 5)

###### 3.2.1.1.2. Workflow Disruptions

Participants emphasised that care delivery workflows were frequently restructured at both task and shift levels due to recurring disruptions. These disruptions stemmed from the need to manage unexpected activities, juggle competing roles and responsibilities, navigate collaboration difficulties, address leadership challenges and respond to communication breakdowns. Unexpected activities included both care‐related and noncare‐related events that were unforeseen by healthcare staff, such as residents or family members urgently requesting to discuss concerns (NM 1), as well as impromptu demands from visitors.

*It is the thing which actually disturbs my work in between, like when visitors come and ask me, ‘where is he?’ or ‘what is he doing now?’ or something like orientation, ‘where is the level three’. I cannot say no to anyone. I should have been doing my medicines and concentrating on it, but we got that situation and act accordingly.* (RN 5)

Beyond providing direct care, nurses are responsible for a wide range of tasks that require them to adopt multiple roles. These include documentation and record‐keeping, care coordination and collaboration, care planning, administrative duties and professional development activities such as research and policy engagement. These nonclinical responsibilities often divert nurses’ attention from hands‐on care, resulting in varied and sometimes delayed care delivery arrangements.

*Ideally, the resident should take priority, but you got other pressures from organisations and governments to complete documentations, have meetings and supervisory and HR, all those things. You have to prioritise what you can and cannot do and sometimes things get missed.* (NM 4)

Collaboration difficulties were reported when coworkers resisted providing assistance (RN 5) or when support from allied health professionals was insufficient.

*You called for a podiatrist since a resident got bunions, but they kept being unavailable for weeks. That puts everything back on us and we can only help the resident by trimming nails without giving him the truly needed.* (NM 1)

Leadership challenges emerged particularly when younger staff members were expected to lead teams comprising older, more experienced colleagues. In some cases, staff also resisted to be reallocated as required by the team leader due to personal relationships with colleagues in the areas where their presence was needed (NM 4).

*Sometimes managers can be really young nurses and they have to direct older care staff. That is really confronting and can cause upset for the rest of the shift. How do they bring their best clinical skills when they have had this big conflict within the team?* (NM 2)

Communication issues further compounded these challenges. Under conditions of time pressure and busyness, staff sometimes failed to engage meaningfully with residents, missing cues that signalled emerging health problems and consequently failing to provide timely care (RN 6). Additionally, ineffective handovers or a perceived lack of necessity to report certain information among team members contributed to communication breakdowns, ultimately disrupting the continuity of care delivery.

*A scheduled appointment of a resident was missed on handover this morning, so we had to stop everything else we are doing, because that person had to be ready at a set time. Everybody else had to wait.* (PCW 5)

*Some carers do not report small things, like changes in mood or appetite, but these signs might mean something. If we do not know, we cannot act.* (RN 4)

###### 3.2.1.1.3. Operational Attributes

Participants described a range of operational attributes that shaped care delivery arrangements, including shift type, facility characteristics, organisational orientation, workplace culture and infrastructure and supply limitations. Morning shifts were identified as having higher workloads than afternoon and night shifts, necessitating more efficient care coordination and prioritisation of time‐sensitive or life‐threatening tasks (PCW 5).

Facility characteristics, such as size, geographic location and explicit regulations, also influenced care planning. As NM 8 noted, in larger settings, longer distances between care areas hinder timely responses to residents’ needs and require staff to rely on the proximity principle, that is, delivering care with whatever resources are immediately available. Facilities located in remote or rural regions face intensified challenges in staffing and support services, including limited access to agency staff, prolonged equipment delivery times and reduced allied health availability. These constraints force healthcare staff to focus on essential care only (RN 1). Although care schedules are typically preplanned at the start of each shift based on routines such as bathing, meals and group activities, participants reported that care is frequently adjusted to accommodate residents’ actual conditions and preferences.

*If somebody is asleep, we do not just go in and wake them up and drag them out of bed and say, ‘you must have a shower and get up’*. *This is a nursing home, not a hospital, and you do not have to, or it is unable to keep everything on pace like planed.* (PCW 1)

Organisational orientation, whether driven by resident‐centred values or financial imperatives, was another key determinant. In facilities with a profit‐driven model, staff reported being pressured to reduce the frequency and duration of care activities such as skincare, which in turn increased the incidence of skin breakdown and pressure injuries (PCW 4). Participants also noted that organisational focus on reportable metrics, such as physical restraints, falls and unplanned weight loss, sometimes led to prioritising care tasks that align with these indicators, and, in some cases, encouraged documentation manipulation to meet performance benchmarks (RN 3).

Workplace culture further influenced care delivery through three observed patterns, involving a hierarchical culture in which healthcare staff deliver care according to managers’ orders (NM 5), an unsafe psychological environment where healthcare staff prioritise delivering visible and quantifiable care due to fear of criticism and punishment (PCW 2) and a divisive culture where healthcare staff refuse to collaborate because of an ‘us versus them’ mentality.

*Sometimes there is an ‘us VS them’ between the registered nurses, or the registered staff and the care staff, particularly in aged care. When people do not work together, residents fall through the cracks. One side assumes the other is doing it, and in the end, it does not get done.* (NM 7)

Lastly, infrastructure and supply limitations were cited as significant impediments to timely care. RN 2 described how failure to restore the overnight gas supply delayed essential morning care routines. Similarly, NM 3 highlighted the unavailability of required clinical resources, such as specialised dressings, due to logistical delays, disrupted the provision of necessary care services.

###### 3.2.1.1.4. Systemic and Contextual Influences

Systemic and contextual factors included public health crises, policies and standards regulated by the governing bodies, inadequate funding and resources and inadequate healthcare information shared between different healthcare settings. Many recalled the COVID‐19 pandemic as a particularly acute period of strategic care rationing, marked by severe shortages in workforce and protective equipment, which left lasting impressions on care delivery experiences.

While participants, especially clinical managers, acknowledged the intent of governing bodies to enhance care quality and safety through more rigorous standards and policies, they also highlighted the unintended consequences of these regulatory frameworks. Specifically, the increasing volume and frequency of mandated reporting requirements significantly expanded the administrative burden placed on RNs in managerial roles. This prevented them from providing hands‐on support to frontline staff during periods of heightened demand, ultimately slowing workflow and, in some cases, creating a perception of disengagement or irresponsibility among team members (NM 4).

Underfunding was also raised as a critical issue by participants, who observed that existing funding arrangements failed to cover the time and resources needed to provide care that met residents’ individual health and well‐being requirements.

*It is not necessarily carers’ fault to not do things properly. They do have a lot of things to do in a very short timeframe. We are not funded to provide exactly the right length of time for the services that we are offering.* (NM 7)

Another challenge centred on the lack of integrated healthcare information systems. Participants described significant difficulties in obtaining timely and comprehensive clinical histories when residents were transferred from hospitals to LTAC settings. Disparate systems and poor communication between sectors often forced staff to ration care based on incomplete information.

*There’s a big difference in how we assess and gain historical information for our consumers. So that might impact providing care at the right time and in the right place when a consumer is transferred from hospital to a nursing home.* (RN 7)

###### 3.2.1.1.5. Resident‐Related Factors

Resident‐related factors shaped care delivery decisions in multiple ways, including residents’ attitudes towards care prioritisation, the complexity of their care needs, individual preferences and the nature of family engagement. Participants consistently reported modifying care arrangements based on residents’ flexibility or rigidity regarding the timing and normal routine of care, particularly when multiple residents required assistance simultaneously.

*Some residents will be very clear and say, ‘No, I want this right now’. But others might see it as a chance to take things differently, saying, ‘I will have breakfast in bed today; you can get me up later’.* (RN 2)

The complexity and acuity of residents’ care needs emerged as a primary driver of prioritisation. Healthcare staff routinely gave precedence to individuals at high risk of falls or skin breakdown and those with existing pressure injuries, terminal conditions, delirium, malnutrition, catheters or multiple chronic diseases (RN 8). Acute clinical events, such as sudden deterioration or psychological crises, were considered nonnegotiable priorities (RN 9). In contrast, residents with lower acuity needs, such as emotional support for loneliness, were often deferred to accommodate more pressing clinical demands (NM 1). However, participants acknowledged that residents who were persistent in voicing their needs, colloquially described as the ‘squeakiest wheel’, were often prioritised to minimise workflow disruptions.

*Sometimes, especially with those that are not the highest need, the squeakiest gets the most care. Some people like to complain more than others that without any great understanding that they are actually reasonably low care compared, but to them, it is their lives.* (NM 2)

Respecting residents’ preferences was viewed as essential for person‐centred care delivery arrangements. Such preferences reflected residents’ desire to maintain autonomy or their reliance on trusted staff for personal care tasks like bathing (PCW 4).

*For some residents, what is important to them is getting to the things they want to do, being with the people they want to be with, not having interventions. [……] Residents themselves will make decisions like, ‘Well, I don’t want to wash my hair today because I want to go and see the choir perform, the children or the animals’.* (RN 9)

Family engagement was also a significant factor in care prioritisation. Participants reported that complaints, particularly from demanding family members, could compel staff to prioritise specific residents in order to prevent escalation to management or regulatory authorities.

*Complaints from families have a huge impact on how you manage your time to care. [……] There is pressure to prioritise that particular resident to appease the family and stop them from all the complaints.* (RN 9)

Conversely, some family members served as informal care partners, offering support with activities such as grooming or meal assistance. This form of constructive engagement helped to alleviate staffing pressures and enhance care delivery, as acknowledged by NM 3.

##### 3.2.1.2. Internal and Subjective Factors Related to ICR in Australian LTAC

Five categories were determined as healthcare staff’s internal and subjective factors which affect the cognitive process and consequences of ICR, involving professional values, physical and psychological unwellness, competence, compounding sociodemographic factors and level of familiarity (Table 3).

**TABLE 3:** Internal and subjective factors.

Categories	Codes
Professional values	Commitment to person‐centred care
	Stick to task‐focused style

Physical and psychological unwellness	Physical inability
	Psychological exhaustion

Competence	Working experience
	Education background
	Emotional intelligence
	Language barrier for CALD healthcare staff
	Leadership competency

Compounding sociodemographic factors	Individual factors
	Workplace‐related factors

Level of familiarity	Familiarity with residents
	Familiarity with coworkers
	Familiarity with facility

Abbreviation: CALD, culturally and linguistically diverse.

###### 3.2.1.2.1. Professional Values

Professional values are the ethical principles, standards and beliefs that guide behaviour, decision‐making and interactions within a professional context [40]. Participants described distinct differences in care delivery arrangements based on the professional values held by healthcare staff, particularly between those who embrace person‐centred care and those who adhere to task‐focused working styles. Healthcare staff committed to person‐centred care prioritised residents’ individual needs and preferences, often demonstrating greater proactivity in care provision. Such practices were perceived as protective, helping to prevent potential harm and functional decline among residents (NM 9). While person‐centredness is widely regarded as an essential component of high‐quality aged care, participants noted that some staff continued to operate within a task‐oriented framework. These individuals tended to focus on completing preassigned duties and responded passively to residents’ requests, reflecting a more transactional and less responsive model of care delivery.

*A lot of carers at aged care are at the degree of task driven. Here is a task. Here is the time to do it. Even though we trained them, they are not thinking of looking outside that task square. They do not have the will to know that one more minute with Mrs Jones, you might be able to identify that something is changing quite dramatically with her.* (RN 8)

###### 3.2.1.2.2. Physical and Psychological Unwellness

When faced with workforce‐related challenges, such as staff shortages and frequent, unpredictable fluctuations, participants commonly reported being physically unable to deliver appropriate care. Physical exhaustion and risk of injury, particularly during extended hours or consecutive shifts, were identified as key barriers to maintaining care quality. These conditions were described as routine in LTAC settings, contributing to a tendency among staff to prioritise only urgent needs and defer less critical tasks to the following shift.

*By the second half of the shift, my body just cannot keep up. I want to do more, but I physically cannot lift or move as quickly anymore, so I leave the low-priority things for the next shift.* (PCW 4)

In addition to physical fatigue, psychological exhaustion also contributed to ICR. When overwhelmed by workload demands or experiencing a sense of being unrecognised and undervalued, healthcare staff reported a diminished capacity to engage emotionally with residents. Under such conditions, they focused solely on completing essential clinical and personal care tasks, often neglecting residents’ emotional and spiritual needs (PCW 6).

###### 3.2.1.2.3. Competence

Competence refers to the ability to perform tasks successfully and efficiently, encompassing a blend of knowledge, skills, judgement and personal attributes that enable individuals to meet established standards within their roles [41]. Participants generally recognised that educational background plays a critical role in shaping care delivery, particularly when comparing RNs who are typically educated to the bachelor level with PCWs, who undergo vocational training. RNs, equipped with more comprehensive and systematic education, were perceived as more capable of identifying early signs of clinical deterioration and initiating appropriate responses. In contrast, PCWs were sometimes described as overlooking subtle clinical cues due to limited training (NM 1). However, several participants argued that practical experience in LTAC settings exerted a greater influence on real‐world competence than formal education. Experience was viewed as essential for cultivating the nuanced, context‐specific skills necessary for effective care delivery, whereas education provided a more theoretical foundation. Newly graduated RNs and those transitioning from acute care settings were frequently characterised as overly task‐focused and insufficiently adaptable to the unique demands of LTAC (NM 3).

Emotional intelligence was also highlighted as a key determinant of competent care delivery. Participants reported that both novice and experienced staff who lacked the emotional capacity to self‐regulate or apply effective communication strategies were more easily distracted by interactions with distressed residents, demanding families or unsupportive colleagues, ultimately impairing judgement and decision‐making of ICR (NM 2). Additionally, communication difficulties stemming from language barriers among culturally and linguistically diverse (CALD) staff were noted to impact their competency to understand residents’ needs and deliver appropriate care.

*It is beautiful when people from the Pacific Islands are coming in and becoming part of our workforce. [……] But the issue that I see is that residents cannot understand them. English is the third or fourth language and the communication is a real issue. Consumers feel like they are treated respectfully with kindness, but the communication is sometimes poor, which then impacts on early escalation of clinical issues.* (NM 3)

Leadership competence emerged as another critical consideration. Participants observed that both novice and seasoned RNs are increasingly being placed in managerial or team leadership roles within LTAC settings. Despite clinical competence, many lacked the leadership training and experience necessary to manage shifts effectively. This leadership gap was reported to compromise shift‐level decision‐making, particularly under high‐pressure or acute conditions (NM 8).

###### 3.2.1.2.4. Compounding Sociodemographic Factors

Participants identified two groups of sociodemographic factors of healthcare staff that influence care delivery arrangements: individual‐level factors, including age, personality, nursing level and cultural background, and workplace‐related factors, such as self‐perceived relationships with coworkers and the primary responsibilities assigned during a shift. Participants aged 40 and above frequently observed generational differences in care approaches, suggesting that younger nurses were sometimes perceived as more detached and less responsive to residents’ needs, such as delaying their response to the call bells (NM 5). Personality traits also played a role in delegation and collaboration. Outgoing and warm‐hearted colleagues were described as more approachable and willing to offer assistance, even when under time pressure (PCW 8). Delegation patterns were also shaped by nursing level, with some RNs choosing to delegate personal care tasks to PCWs even when they responded to residents’ call bells themselves, believing such tasks fell outside their scope (PCW 3).

Cultural background exerted both positive and negative influences on care delivery. Participants from cultures that emphasise respect for older people often displayed stronger person‐centred values. However, cultural differences occasionally led to workplace tensions, including exclusionary behaviour and poor integration, which undermined both team cohesion and the quality of resident interactions.

*Cultural differences between staff, and between staff and residents, also matter. People from the same culture that work together can exclude other cultures, speaking their own language over top and not interacting with residents.* (RN 7)

In terms of workplace‐related factors, self‐perceived relationships with coworkers strongly influenced the delegation and redistribution of care tasks. PCW 7 noted that she preferred to request assistance from colleagues with whom she had established reciprocal support, often delegating simpler tasks to maintain those positive relationships. Conversely, participants reported reluctance to be reallocated to areas with unfamiliar or unfriendly coworkers, even when workforce shortages demanded redistribution (RN 9). Several participants also noted that the primary responsibilities assigned during a shift shaped care priorities. RN 3 explained that when assuming managerial duties, she would prioritise nursing documentation over direct care. Similarly, PCWs who had completed diploma‐level training in medication administration reported prioritising medication rounds over personal care tasks (PCW 9).

###### 3.2.1.2.5. Level of Familiarity

Participants highlighted that familiarity significantly influences care delivery arrangements across three key dimensions: familiarity with residents, coworkers and the facility. These forms of familiarity were considered as essential for promoting safe, efficient and responsive care. Participants consistently emphasised that such familiarity could only be developed through being a consistent and regular staff member in a LTAC setting, rather than through casual or agency work. Familiarity with residents was considered a foundational element for delivering person‐centred care. Participants stressed the importance of understanding residents’ health conditions, behavioural patterns, preferences and daily routines, which enabled them to anticipate care needs, prioritise effectively and respond proactively to emerging risk.

*As a care worker, you know about your residents, so you know who wants to get up first in the morning and what time they get up. [… …] If there is a buzzer going off the person that has a fall risk, you would go and attend to them first and do everything else after that.* (PCW 2)

Familiarity with coworkers influenced the delegation and redistribution of care tasks. Participants explained that knowing a colleague’s competencies and working style fostered confidence in delegating tasks, particularly those requiring clinical judgement or specialised skills. In contrast, unfamiliarity often led to reluctance or the delegation of only low‐complexity tasks.

*If you know what they can do and you have worked with them before, you are more confident to delegate properly. If not, you hesitate or only ask them to do simple things.* (PCW 7)

Familiarity with the facility, including the physical layout, availability of resources and internal protocols, was also seen as facilitating more timely responses and efficient coordination during routine tasks or emergency situations. Participants noted that such operational knowledge reduces delays and enhances team cohesion during high‐pressure shifts (RN 1).

#### 3.2.2. The Decision‐Making Process and Outcomes of ICR in Australian LTAC

**FIGURE 2:**
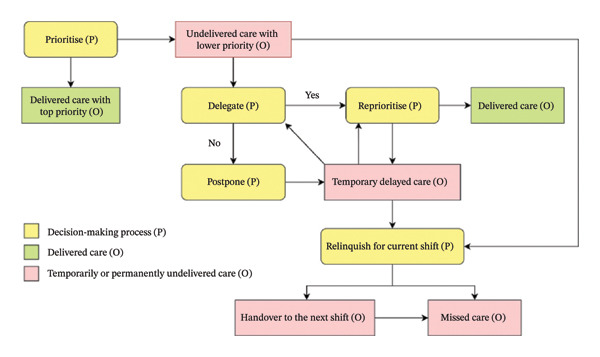
Diagram of the cognitive process and outcomes of ICR in Australian LTAC. “P” for cognitive process and “O” for care delivery outcomes.

Under the influence of various external and internal factors, healthcare staff were found to engage in a dynamic cognitive process during ICR that involves prioritisation, delegation, reprioritisation, postponement and, when necessary, relinquishment of care for the current shift, leading to real‐time care delivery outcomes (Figure 2). This pathway was derived from recurring patterns in participants’ accounts of multitasking on ‘busy’ or ‘hectic’ shifts. They described beginning by organising competing care tasks, then allocating some tasks to coworkers and repeatedly adjusting which activities could be completed or deferred as circumstances changed. At the same time, they anticipated and often accepted that certain care would not be completed within that shift due to ongoing resource constraints.

Participants consistently described prioritisation as a deliberate and necessary first step in rationing care. Within this framework, prioritisation refers to how healthcare staff order competing care demands. In practice, this involves delivering care as scheduled by the team leader (e.g., morning routines and medication rounds) while also responding promptly to emergent needs, such as call bells and acute clinical changes. Most participants reported that upon hearing call bells, they would communicate with each resident to triage their needs before determining the order of response. However, one PCW described using a more linear strategy, adopting a ‘first come, first serve’ approach when responding to ringing bells (PCW 5). Once care needs are prioritised, those assigned the highest priority are delivered immediately due to their time‐sensitive or life‐threatening nature.

Following prioritisation, delegation would be adopted to ensure a timely response to residents’ low‐priority needs through teamwork with coworkers. If delegation was successful, delegators would focus on care with highest priority and mentally revise their ‘to‐do list’ for the remaining care with lower priority to be addressed later in the shift.

*What I usually do is make sure I have delegated tasks first after prioritisation. I check that the care staff have been given clear, adequate instructions so they know what they need to do.* (NM 6)

By contrast, postponement would follow when delegation was not possible because colleagues were unavailable or already overloaded, with staff intending to return to low‐priority care later in the shift. When conditions (e.g., time) allowed or help became available, staff would reprioritise or delegate the temporarily delayed care to ensure it was completed. For instance, RN 1 described a case of delayed care delivery by delegation in which a resident requested a cushion during her medication round. Since all PCWs were occupied, she assured the resident that she would provide the cushion after completing the round. Later, while continuing the round, RN 1 encountered a PCW who had just finished another task, so she delegated the request, and the PCW carried it out immediately. Yet, as one advisory group member noted, successful delegation did not always guarantee care delivery:

*You might delegate something, thinking it will get done, but sometimes it does not happen. You check at the end of the shift and realise it was missed, even though you thought you had handed it over properly.* (G1‐1‐4)

When workload pressures persisted, relinquishment would occur, referring to a decision that certain care would not be delivered within the current shift. This happened when the iterative ‘delegation/reprioritisation/postponement’ cycle could no longer accommodate low‐priority care needs, particularly when high‐priority care continued to accumulate and opportunities for teamwork are limited or absent. Under these circumstances, low‐priority care remained at the bottom of the list and was either handed over to the next shift or became missed care, in that they are never delivered within an acceptable time frame. This situation was particularly evident for needs considered less urgent or difficult to measure, such as emotional or psychosocial support (RN 8). Participants generally did not perceive the act of handing over care as an indication of missed care. They emphasised that care in LTAC is continuous, and that expecting every task to be completed within a single shift is unrealistic given the potential for unexpected fluctuations in workload and acuity. As one nurse manager explained:

*Anything can happen on any kind of shift, and it is not fair to say everything should be done by a single shift. Nursing homes open 24 h, 7 days and we are supposed to pass things on.* (NM 9)

Similarly, a PCW commented that:

*Because I am here five days a week, if I have not done it the day before, I am going to do it the next day. I do not think of that as being missed because it was eventually delivered. It is more like being delayed*. (PCW 3)

In more extreme situations, care relinquishment occurred earlier, particularly under conditions of severe workforce shortages or limited resources, as noted by one advisory group participant:

*Sometimes the clinical manager has to decide at the beginning of the shift what care will not happen today, because we just do not have the staff or resources to make it possible.* (G2‐1‐3)

Participants highlighted that determining when to provide care is only one dimension of the outcomes shaped by ICR. Healthcare staff are also required to make real‐time decisions about the level of completeness with which care is delivered. These decisions range from delivering care in a standard and thorough manner, to rushing care in a substandard way or substituting the original care with a simplified version, often in response to time pressures and workforce constraints. Many participants acknowledged that, under the cognitive process of ICR, care is frequently rushed or replaced. One RN (RN 2) described a situation where she would hastily serve meals to a resident before responding to a call bell from another resident who might have higher needs when there was no one to help, although she knew that resident had not properly consumed the meal. A PCW (PCW 4) reported that if a resident was scheduled for a shower during a particularly busy shift but required two‐person assistance which would consume significant time, she would instead provide a proper wash to prevent falling behind schedule. Participants also emphasised that the order and completeness of care delivery are not necessarily parallel. Prioritised care is not always guaranteed to be delivered in a standard or complete manner, while less urgent care may be carried out more thoroughly when time allows.

*Just because something is prioritised does not mean it gets done perfectly. Sometimes you rush through it because it is urgent. And oddly, the less urgent things might be done more thoroughly if you happen to have the time.* (RN 7)

Particularly, participants mentioned that their judgement throughout the cognitive process of ICR is guided by two principles, including risk‐averse, consequence‐focused mindset and trade‐offs between task‐focused and person‐centred approaches. They explained that, over time, care delivery in LTAC settings becomes relatively routine and stable, even when managing emergent situations. As a result, most decisions are made intuitively, drawing on accumulated work experience rather than critically engaging with formal knowledge or training. This tendency becomes more pronounced under time pressure, where rapid decision‐making is necessary. Healthcare staff would first consider the safety in arranging care delivery by avoiding risks and negative consequences to residents and themselves if the care services were delayed or missed.

*It is more risk-based, like if I do not do this now, what is going to be the consequences? What is the risk for the person? What is the risk for my relationships with colleagues? If I am continually not completing my tasks, they are not going to want to work with me. Or if I do not do this, am I going to get yelled at by the manager or even lose my job?* (NM 4)

Although participants expressed a strong desire to provide high‐quality, person‐centred care, they also acknowledged the need to balance this ideal with the practical demands of completing scheduled tasks. Maintaining this balance often required them to navigate tensions between respecting residents’ overall well‐being, preferences and autonomy and meeting the efficiency demands of the shift. Participants noted that person‐centred care is inherently more time‐consuming, as it relies on building deep understanding through frequent and sustained interactions. In contrast, a task‐focused approach was seen as more efficient, particularly under time pressure or in emergency situations, allowing staff to complete essential care activities more quickly. When engaging in ICR, healthcare staff described making continuous trade‐offs along a spectrum, with one end representing pure person‐centredness and the other pure task orientation.

*You need to constantly be juggling. ‘Do I stop and listen and talk to residents?’, or ‘Do I push through the list?’ There is always a trade-off between ideal and realistic. So, when we have to be task-focused, we try to weave in small moments, like chatting while giving meds or doing skincare, to keep that person-centredness.* (RN 4)

## 4. Discussion

This study developed a grounded theoretical framework that explicates the underlying logic and shift‐level cognitive pathway of ICR in Australian LTAC. Moving beyond prevailing conceptualisations that equate ICR with care omission, the findings demonstrate that rationing is better understood as a dynamic sequencing process through which healthcare staff strategically organise the order and completeness of care under resource constraints. By integrating structural factors, iterative decision cycles and guiding principles of risk appraisal and trade‐off reasoning, the framework offers a mechanism‐based explanation of how rationing unfolds in everyday practice. While grounded in the organisational and regulatory characteristics of Australian LTAC, the framework advances theoretical clarity regarding the distinction between ICR and missed care and provides a coherent analytical basis for examining care quality and workforce decision‐making in other resource‐constrained healthcare environments.

### 4.1. Contextual Factors Shaping the Operation of ICR in Australian LTAC

Australian LTAC settings are characterised by more severe workforce shortages, a highly diversified workforce composition and a resident‐led care paradigm in contrast to acute clinical settings. These contextual features serve as critical drivers behind the contributing factors of ICR identified in this study.

Workforce shortages are a well‐established contributor to missed care across various healthcare settings, with improving recruitment and retention of healthcare staff being identified as an ultimate solution [42]. However, staffing challenges in Australian LTAC are particularly acute. LTAC is often perceived as a sector marked by low pay, limited career progression, low social recognition and minimal professional fulfilment, all of which deter prospective workers [16]. Concurrently, the increasing complexity of residents’ health conditions contributes to an overwhelming workload, which exacerbates burnout, turnover and staff attrition [43]. To address this workforce crisis, Australian governments have introduced a range of incentive‐based policies aimed at filling workforce gaps, attracting nursing students from educational institutions, healthcare professionals from other sectors, agency staff and overseas‐trained workers [44]. However, as highlighted by the current study, these recruitment strategies often address staffing numbers without ensuring the necessary competencies to deliver high‐quality care in LTAC settings [45]. Many recruits lack the contextual knowledge and practical skills that can only be acquired through extensive experience in the LTAC sector. Consequently, experienced staff are burdened with the dual task of supervising novices while meeting their own responsibilities, further stretching care capacity. Therefore, in addition to increasing staff numbers, it is imperative to develop strategies that accelerate competence without disrupting day‐to‐day operations [43]. Simulation‐based training, role‐specific workshops and coursework tailored to the practical realities of LTAC could expedite capability development and support workforce sustainability [46].

Alongside staffing challenges, this study identified an inharmonious workplace culture, stemming from a highly diversified workforce in terms of qualifications, cultural backgrounds and generational experiences, as a significant contributor to ICR. In LTAC, RNs and PCWs work collaboratively, with PCWs making up the majority of the workforce [27]. However, existing research often overlooks the distinct role boundaries and division of responsibilities in these settings by primarily capturing nurses’ perspectives on missed care. A notable finding of this study is that RNs may not perceive undelivered personal care as missed care when such tasks have been delegated to PCWs. Although RNs are primarily responsible for clinical care and medication administration, they are also expected to assist with personal care when PCWs are overwhelmed. However, some RNs were described as adopting a ‘none‐of‐my‐business’ mindset, viewing personal care as beneath their qualifications [23]. This attitude reinforces role‐based tensions, weakens collaboration and erodes mutual trust, ultimately increasing the risk of care delays and omissions [47].

Cultural diversity, while valuable for promoting culturally responsive care, also introduces complexities in care delivery and team dynamics [48]. As employment in LTAC settings has become a common pathway for obtaining working visas and permanent residency [43], cultural heterogeneity in the workforce has intensified. Participants reported instances where care delivery was compromised due to implicit bias or reluctance to assist colleagues or residents from different cultural backgrounds [49]. These divisive subcultures, shaped by cultural tension, warrant further investigation, as they can significantly disrupt team cohesion and care continuity [50].

Generational friction was also observed, particularly between academically qualified novice leaders and experienced frontline staff. Although previous literature [51] emphasised that younger leaders might lack the experience and competency to manage care rosters effectively, this study highlights a complementary perspective which is that resistance from staff towards new leaders may stem from relational rather than competency‐based concerns. Multigenerational nursing teams were described as prone to conflict and miscommunication in the absence of proactive management strategies [52]. Value clashes across age cohorts were found to destabilise cooperation, with staff cooperation closely linked to relational trust [52]. Specifically, cognitive trust in supervisors correlated with improved functional performance, while distrust led to participation reluctance and reductions in work quality [53].

To foster a more collaborative work environment, it is essential to complement leadership training with mechanisms that build mutual trust, such as regular debriefing, structured feedback and open communication between leaders and team members [37]. This approach could also help ease tensions between staff of different qualifications [47] and cultural backgrounds [50]. Furthermore, strengthening professional values across the workforce may enhance cooperation and compliance with care delivery practices, even within a highly diverse team composition [40].

Finally, this study reinforces the importance of a resident‐oriented care paradigm that plays a pivotal role in facilitating person‐centred care planning within LTAC settings, which is particularly dependent on healthcare staff’s familiarity with residents’ care needs and preferences. This finding aligns with the Strengthened Aged Care Quality Standards [26], which explicitly mandate that high‐quality, person‐centred care must be tailored to each resident’s needs, goals and preferences, with respect for their identity, culture, life experiences and autonomy. However, achieving true person‐centred care remains a challenge in LTAC settings, not only due to limited time and resources but also due to a task‐focused mindset [54] and the workforce instability that impairs continuity and familiarity [23]. Given the limited feasibility of rapidly increasing time and resources, attention should be directed towards more modifiable barriers. These include strengthening staff commitment to person‐centred care through targeted training and fostering an organisational culture that rewards attitudinal change [54]. For staff resistant to change due to entrenched beliefs, leadership should prioritise value transformation as a key component of team development [55]. Additionally, while agency staff may be essential to addressing workforce shortages [56], facilities should seek to stabilise their allocations and rotate permanent staff judiciously. Comprehensive, standardised handovers, covering not only clinical information but also psychosocial and emotional insights, should be implemented to promote continuity and uphold person‐centred care principles [57].

### 4.2. An Evolution of the Theoretical Framework of ICR

The framework developed in this study advances the conceptualisation of ICR by clarifying its underlying logic and internal processual structure within Australian LTAC. While Schubert conceptualised implicit rationing primarily as the withholding or nonprovision of necessary nursing care under resource constraints [17], the present findings indicated that healthcare staff do not generally approach rationing with an intention of omitting care. Only under extreme resource scarcity do staff judge some care delivery to be unfeasible at the outset. More commonly, omissions occur after efforts to maintain care delivery, such as reprioritisation and delegation, have been exhausted. In this sense, ICR functions as an adaptive effort oriented not primarily towards withholding care, but towards preserving safe and functional care delivery within limited resources. This reframing shifts the analytical focus from care left undone to the strategic organisation of care that precedes, mitigates or sometimes prevents omissions and their potentially detrimental outcomes [25].

Building on this logic, the framework conceptualises ICR not as a discrete event but as a dynamic, shift‐level decision‐making process. This processual orientation provides a more explanatory account of how ICR unfolds over time, particularly in environments characterised by instability and competing priorities. As demonstrated in the Findings, healthcare staff move iteratively through cycles of prioritisation, delegation, reprioritisation and postponement. Relinquishment arises when care delivery can no longer be secured within acceptable circumstances, either after repeated efforts at reorganisation or when feasibility is constrained from the outset. By explicating this pathway, the framework distinguishes the cognitive mechanism of ICR from its observable care delivery outcomes. Within this process, care delivery may occur in different sequences and with varying degrees of completeness, including immediate delivery, delayed delivery, delegated delivery or delivery in a less complete before care is ultimately omitted. Missed care is therefore conceptualised as one potential consequence of this broader process rather than its defining feature. This distinction refines the conceptual boundaries between ICR and missed care, which have often been used interchangeably in previous literature [18], and may support greater conceptual rigour and consistency in future research addressing related phenomena.

Within this multidimensional outcome space of ICR, delayed care and missed care warrant careful conceptual differentiation. The present framework illustrates how delayed care may be completed later through ongoing efforts to preserve care delivery, or, alternatively transition into missed care when delivery can no longer be secured within a shift. Importantly, the boundary between delayed and missed care in LTAC should not be understood simply as a single‐shift temporal cutoff. Rather, it depends on whether ongoing efforts to reorganise care delivery can still secure completion within an acceptable timeframe for the particular care activity. However, this acceptable timeframe is unlikely to be uniform across care domains, reflecting differences in clinical urgency and acceptable risk tolerance [18]. For time‐sensitive and high‐risk care, such as medication administration or toileting assistance, the acceptable window for delay may be measured in minutes, beyond which delayed care will rapidly become missed care, even within a single shift [17]. By contrast, for less time‐bounded and low‐risk care, omissions within the current shift may still be addressed in subsequent shifts or days. In such cases, healthcare staff may interpret these omissions as still falling within an acceptable period of delay rather than as missed care, as suggested by the Findings. Moreover, the longer acceptable timeframe for deferring some care reflects not only lower urgency and risk but also the person‐centred nature of LTAC. When conditions allow, care delivery is expected to remain flexible to accommodate residents’ routines, preferences and readiness, rather than being strictly governed by shift‐bound schedules [23]. This differs from acute clinical settings, where care is more often organised around time‐critical interventions and tightly structured workflows, making the boundary between delays and omissions comparatively more fixed than in LTAC [15]. Accordingly, the flexible boundary between delayed and missed care should not be interpreted as conceptual ambiguity within the framework, but as a defining contextual characteristic of LTAC practice.

Beyond identifying the cognitive stages and related care delivery outcomes, interpreting this framework also requires attention to transitions across these stages within the cognitive pathway of ICR. Two interrelated logics were identified as guiding the cognitive transitions: a risk‐averse, consequence‐focused orientation and trade‐offs between person‐centred and task‐focused approaches to care delivery. The risk‐averse, consequence‐focused orientation guided healthcare staff to anticipate potential clinical, relational and professional consequences to protect the safety of care practice. Meanwhile, the trade‐off logic helped healthcare staff to preserve care quality brought by person‐centredness while maintaining the care efficiency afforded by task‐focused approaches. These logics appeared to function as heuristics [51], that is, practical mental shortcuts used to support ‘good‐enough’ decision‐making when the ability to reach optimal solutions is constrained. These heuristics evolved through repeated exposure to routine yet resource‐constrained LTAC environments and informed habitual judgements in daily practice. Rather than appraising each influencing factor separately, healthcare staff applied these practical logics to make rapid, holistic judgements about how multiple internal and external influences combined to affect the safety, quality and efficiency of care delivery. This interpretation is consistent with the Practice‐Primed Decision Model [58], which conceptualises nursing decision‐making in time‐limited, high‐stakes environments as shaped by experience‐based judgement, pattern recognition, situation awareness and multiple interacting contextual influences, rather than by single factors operating independently. By conceptualising ICR decisions as the product of compound, context‐sensitive recalibrations, the present framework helps explain why similar influencing factors may produce different cognitive transitions and care delivery outcomes across shifts, teams and individuals [51].

In summary, although the framework was developed within Australian LTAC, its transferability is most plausible at the level of the cognitive pathway and its guiding principles. This is supported by the widespread presence of structural pressures across healthcare systems, such as workforce shortages, workflow disruption and limitations in leadership and teamwork capacity [15]. In particular, the broader applicability of the cognitive pathway is indicated by a grounded theory study in which ICU nurses in China were found to engage in a similarly adaptive decision‐making process around missed nursing care, involving setting priorities, seeking help, delaying care and omitting care [25]. Regarding the guiding principles, a study in Finnish nursing homes [59] and review evidence from hospital and home‐care settings [60] also suggest that staff decision‐making on prioritisation and missed nursing care under resource constraints often involves balancing competing demands related to care safety, care quality and workflow efficiency. Taken together, the potential transferability of this framework could offer a useful analytic lens for understanding how ICR unfolds in other constrained care settings and national contexts. However, the specific factors that activate or shape these processes are likely to remain context dependent. As discussed earlier, several external and internal factors identified within the current framework are further embedded within the structural and regulatory characteristics of Australian LTAC, including funding arrangements, reliance on agency staffing and multicultural workforce dynamics. Accordingly, the specific triggers and manifestations of ICR are likely to be shaped by regional regulatory frameworks, organisational cultures, staffing models and workforce characteristics. Therefore, when applying or empirically examining this framework in other settings, careful attention should be paid to contextual configurations that may modify how the mechanism operates in practice.

### 4.3. Limitations

Although multiple strategies were adopted to ensure rigour and trustworthiness of the development of the theoretical framework, this study failed to apply theoretical sampling as recommended in the SGT approach. Theoretical sampling enables researchers to refine developing categories, explore negative cases and reach conceptual saturation more systematically [33]. In the current study, however, participants were recruited in advance within each phase using purposive and convenience strategies, rather than being selected iteratively during data collection based on emerging theoretical needs. Nonetheless, the data collection process remained analytically responsive. Each interview was analysed immediately after completion, and the interview guide was iteratively adjusted to address emerging concepts and theoretical questions. This allowed subsequent participants to contribute insights that extended or clarified the evolving framework. Furthermore, although the sample was fixed within each phase, the preliminary theoretical framework was evaluated by advisory groups that comprised participants from different settings and states. Their validation of the framework as both accurate and comprehensive in reflecting their own empirical experiences helped confirm the credibility and conceptual robustness of the findings.

A further consideration is that some moral–professional influences discussed in the broader literature, such as moral reasoning, professional identity and perceived accountability, were not explicitly identified as standalone factors shaping the ICR pathway. Although some participants described reflecting on or ruminating over ethical and professional dilemmas arising from ICR, these reflections appeared more often as responses to constrained circumstances than as clearly articulated influences in the current framework. Participants conveyed that, despite the moral and emotional discomfort, they believed they had done their best within the limits of the circumstances and had little capacity to alter the situation further. Therefore, the limited salience of these constructs in the present data does not indicate a lack of theoretical saturation. Rather, the present findings suggest that such influences may have limited or context‐dependent effects on ICR in Australian LTAC, at least as perceived by the participants included in this study. However, emerging evidence indicates that related constructs, including professional values and moral sensitivity [40], are associated with missed nursing care, suggesting that moral–professional influences may still be relevant to care rationing processes more broadly. Further research using samples drawn from more diverse backgrounds or other methodological approaches is therefore required to examine whether and how these influences shape ICR across different contexts.

## 5. Conclusion

This study developed a grounded theoretical framework that reconceptualises ICR as a dynamic, shift‐level sequencing mechanism rather than a mere manifestation of care omission. By explicating the iterative cognitive pathway and identifying the guiding principles that regulate its progression, the framework advances a mechanism‐based explanation of how rationing decisions unfold in everyday LTAC practice. Importantly, the findings clarify the conceptual distinction between ICR as a cognitive process and missed care as one potential outcome within a broader multidimensional outcome space defined by the order and completeness of care delivery. This distinction enhances theoretical precision and supports greater rigour in future measurement and intervention research aimed at optimising care delivery under resource constraints. Future research should examine how this ICR pathway operates across diverse healthcare environments and explore its application in instrument development, workforce strategy and quality improvement initiatives designed to mitigating the unintended consequences of ICR decisions.

## Funding

This work was supported by the 2023 Telstra‐UOW Hub for Artificial Intelligence of Things (AIOT) Solutions Seed Funding Program (Project Number M2253‐1). Open access publishing facilitated by University of Wollongong, as part of the Wiley ‐ University of Wollongong agreement via the Council of Australasian University Librarians.

## Conflicts of Interest

The authors declare no conflicts of interest.

## Supporting Information

Additional supporting information can be found online in the Supporting Information section.

## Supporting information


**Supporting Information 1** The Consolidated Criteria for Reporting Qualitative Research (COREQ) Checklist. This checklist is provided to facilitate the identification of essential methodological information and supports the reproducibility and credibility of the study. It was referenced in the Methods section, where the reporting of this study was noted to align with both the Standards for Reporting Qualitative Research (SRQR) and the COREQ checklist. The checklist enables readers’ appraisal of the methodological transparency of this study.

**Supporting Information 2** Interview Guides. This file contains the initial interview guides employed during Phase 1 of data collection, including the semistructured interview guides for registered nurses and managers and the structured question set for personal care workers. Details regarding the development, pilot testing and subsequent revisions of the interview guides are provided in the Data Collection section of the​ manuscript.

## Data Availability

The data that support the findings of this study are available from the corresponding author upon reasonable request.
