# Supplementary material for: A Theoretical Framework of Implicit Care Rationing in Australian Long‐Term Aged Care Settings: A Straussian Grounded Theory Study
Source: J Nurs Manag. 2026 Apr 29;2026:8676600. doi: 10.1155/jonm/8676600 (PMC13129217; doi:10.1155/jonm/8676600)
Supplement: Supplementary file 1 — Supporting Information 1 The Consolidated Criteria for Reporting Qualitative Research (COREQ) Checklist. This checklist is provided to facilitate the identification of essential methodological information and supports the reproducibility and credibility of the study. It was referenced in the Methods section, where the reporting of this study was noted to align with both the Standards for Reporting Qualitative Research (SRQR) and the COREQ checklist. The checklist enables readers’ appraisal of the methodological transparency of this study. [file JONM-2026-8676600-s002.pdf]

COREQ (COnsolidated criteria for REporting Qualitative research) Checklist

A checklist of items that should be included in reports of qualitative research. You must report the page number in your manuscript where you consider each of the items listed in this checklist. If you have not included this information, either revise your manuscript accordingly before submitting or note N/A.

Topic	Item No.	Guide Questions/Description	Reported on Page No.
**Domain 1: Research team and reflexivity**			
Personal characteristics			
Interviewer/facilitator	1	Which author/s conducted the interview or focus group?	P11
Credentials	2	What were the researcher's credentials? E.g. PhD, MD	P11
Occupation	3	What was their occupation at the time of the study?	P10, P11
Gender	4	Was the researcher male or female?	P11
Experience and training	5	What experience or training did the researcher have?	P11
Relationship with participants			
Relationship established	6	Was a relationship established prior to study commencement?	P10, P11
Participant knowledge of the interviewer	7	What did the participants know about the researcher? e.g. personal goals, reasons for doing the research	P10, P11
Interviewer characteristics	8	What characteristics were reported about the inter viewer/facilitator? e.g. Bias, assumptions, reasons and interests in the research topic	P11
**Domain 2: Study design**			
Theoretical framework			
Methodological orientation and Theory	9	What methodological orientation was stated to underpin the study? e.g. grounded theory, discourse analysis, ethnography, phenomenology, content analysis	P8 - P9
Participant selection			
Sampling	10	How were participants selected? e.g. purposive, convenience, consecutive, snowball	P9 - P10
Method of approach	11	How were participants approached? e.g. face-to-face, telephone, mail, email	P9 - P10
Sample size	12	How many participants were in the study?	P15- P17
Non-participation	13	How many people refused to participate or dropped out? Reasons?	P16
Setting			
Setting of data collection	14	Where was the data collected? e.g. home, clinic, workplace	P 16 - P18
Presence of non-participants	15	Was anyone else present besides the participants and researchers?	P16 - P18
Description of sample	16	What are the important characteristics of the sample? e.g. demographic data, date	P15 - P17
Data collection			
Interview guide	17	Were questions, prompts, guides provided by the authors? Was it pilot tested?	P11
Repeat interviews	18	Were repeat inter views carried out? If yes, how many?	P16
Audio/visual recording	19	Did the research use audio or visual recording to collect the data?	P11 - P13
Field notes	20	Were field notes made during and/or after the inter view or focus group?	P11
Duration	21	What was the duration of the inter views or focus group?	P15, P16
Data saturation	22	Was data saturation discussed?	P16
Transcripts returned	23	Were transcripts returned to participants for comment and/or	P16

Topic	Item No.	Guide Questions/Description	Reported on Page No.
		correction?	
**Domain 3: analysis and findings**			
Data analysis			
Number of data coders	24	How many data coders coded the data?	P13
Description of the coding tree	25	Did authors provide a description of the coding tree?	P18, P27
Derivation of themes	26	Were themes identified in advance or derived from the data?	P14
Software	27	What software, if applicable, was used to manage the data?	P13
Participant checking	28	Did participants provide feedback on the findings?	P16 - P17
Reporting			
Quotations presented	29	Were participant quotations presented to illustrate the themes/findings? Was each quotation identified? e.g. participant number	P18 -P37
Data and findings consistent	30	Was there consistency between the data presented and the findings?	P18 -P37
Clarity of major themes	31	Were major themes clearly presented in the findings?	P18 -P37
Clarity of minor themes	32	Is there a description of diverse cases or discussion of minor themes?	P18 -P37

Developed from: Tong A, Sainsbury P, Craig J. Consolidated criteria for reporting qualitative research (COREQ): a 32-item checklist for interviews and focus groups. *International Journal for Quality in Health Care*. 2007. Volume 19, Number 6: pp. 349 – 357
