# Supplementary material for: A Theoretical Framework of Implicit Care Rationing in Australian Long‐Term Aged Care Settings: A Straussian Grounded Theory Study
Source: J Nurs Manag. 2026 Apr 29;2026:8676600. doi: 10.1155/jonm/8676600 (PMC13129217; doi:10.1155/jonm/8676600)
Supplement: Supplementary file 2 — Supporting Information 2 Interview Guides. This file contains the initial interview guides employed during Phase 1 of data collection, including the semistructured interview guides for registered nurses and managers and the structured question set for personal care workers. Details regarding the development, pilot testing and subsequent revisions of the interview guides are provided in the Data Collection section of the​ manuscript. [file JONM-2026-8676600-s001.pdf]

Semi-Structured Interview Guide for Nurses and Managers

Opening Questions

- Could you please briefly introduce yourself, perhaps including some basic information about your workplace and share your daily work responsibility and routine?
- Could you tell me about your understanding of implicit care rationing within RACFs is?
- Have you ever experienced or heard about instances of implicit care rationing from colleagues' reports or residents' and family members' complaints within your facility? If so, could you share some examples?
- Can you see patterns of implicitly rationed care regarding to your current roles and responsibilities?
- Are there any differences of implicit rationing patterns between different roles, for instance, RNs VS PCWs, and mangers VS hands-on care staff?

Structure

- What factors, considerations or criteria you perceive could influence healthcare workers' decisions on implicit care rationing?

  PROMPT: Could you describe any structural elements (such as policies, staffing levels and resources, or individual characteristics) that you believe contribute to implicit care rationing in RACFs?

- What factors do you believe to be the root causes to the happens of implicit care rationing?

Process

- What’s your actual decision-making process when it comes to implicit care rationing?

  PROMT: What are you based on when making these decisions? Experiences? Habits? Knowledge? Instincts?

- When you make these decisions, will you discuss with others? If so, who will you go for suggestions and opinions?

- Normally, prioritisation are conscious decisions, would you also be conscious about your decisions when delaying or omitting care activities?

Outcomes

- If you are a manger, are your able to recognise the decision of implicit care rationing made by other colleagues and co-workers, especially those who have different responsibilities?
- How could you assess the impacts and effectiveness of your (and others') implicit care rationing decisions that lead to tangible and intangible outcomes?
- In your opinion, who do you think is affected by decisions related to implicit care rationing, and how do you think it impacts them?
- Do you and your colleagues take time to reflect on rationing decisions to identify and reduce any avoidable instances of care rationing?
- Are there any strategies or practices you've encountered or would recommend to effectively address implicit care rationing challenges?

Closing Question

- Is there anything else you would like to share about your experiences or insights related to implicit rationing of care in residential aged care?

Structured Interview Questions for Personal Care Workers

1. Could you walk me through your typical workday as a personal care worker in a residential aged care facility, both for day and night shifts?
2. Is your daily routine strictly set by the manager, or do you adapt it to cater to real-world situations?

   Prompt: If you make adjustments, what kinds of circumstances lead you to change the routine?
3. Imagine a situation where you're the only one available, and several residents need care simultaneously—how do you decide what to do first and what can be delayed for a while?

   Prompt: What changes when other personal care workers are available to help in such situations?

   Prompt: In turn, if a co-worker, such as another personal care worker or a nurse, asks for your help, what factors influence whether you assist with their tasks?
4. If the previous shift didn't complete certain care services for your residents, would you address those services? Why or why not?
5. How do you feel when you can't provide all the care services your customers need?

   Prompt: Do you share these feelings with colleagues? Why or why not?

   Prompt: How do these feelings influence your decisions in future care scenarios?
6. In your view, what could be improved to enhance the quality and effectiveness of care services provided by personal care workers?

Please note that this guide only represents the main themes to be discussed with the participants and as such does not include the various prompts that may also be used (examples given for each question). Non-leading and general prompts will also be used, such as "Can you please tell me a little bit more about that?" and "What does that look like for you".

In accordance with the Straussian Grounded Theory approach to data collection, the interview guide will continue to evolve throughout the research process, informed by the ongoing analysis and emerging insights
